# Scoping Renal Cell Carcinoma in the Rectal and Anal Canal: A Literature Review

**DOI:** 10.7759/cureus.6330

**Published:** 2019-12-09

**Authors:** Shamsuddin M Anwar, Deepak P Kalbi, Astha Upadhyaya, Anum Aqsa, Indraneil Mukherjee

**Affiliations:** 1 Internal Medicine, Staten Island University Hospital, Staten Island, USA; 2 Internal Medicine, Northwell Health, New York, USA; 3 Internal Medicine, Odessa National Medical University, Odessa, UKR; 4 Surgery, Staten Island University Hospital, Staten Island, USA

**Keywords:** renal cell carcinoma, metastasis, metastatic anal cancer

## Abstract

Renal cell carcinoma (RCC) is a common tumor of the kidneys that can often metastasize to other organs, including the lungs, brain, bones, and adrenal glands. However, colon involvement is less common, with metastasis to the rectum and anal canal being an extremely rare phenomenon. The present study describes patients with renal cell carcinoma metastasizing to this unusual location in the gastrointestinal tract (GI).

## Introduction and background

Renal cell carcinoma (RCC) is a condition that is preferably treated by surgical resection, especially in patients without metastasis. Following resection, patients should be carefully monitored for relapse and recurrence. Current guidelines do not recommend adjuvant therapy or immunotherapy after resection of the primary tumor.

Surveillance recommendations following resection are usually tailored to individual patients and depend on the degree of tumor node involvement and metastasis at the time of resection. Although no randomized trials have shown that surveillance after definitive therapy provides survival benefits, surveillance protocols focus on evaluating sites that are frequently involved in metastasis, including the lungs, liver, renal fossa, and bones. Generally, surveillance includes frequent physical examination; laboratory testing that includes urinalysis and concentrations of blood urea nitrogen and creatinine; and imaging modalities, including chest X-ray, CT of the chest and abdomen, ultrasound, and MRI, depending on individual patients. Specific biomarkers may play a promising role in the surveillance and early diagnosis, but the utility of these tests has not yet been determined. 

## Review

We searched the PubMed database using the keywords “renal cell carcinoma”, “rectum”, and “metastasis,” for articles and case reports published through November 2019. Additional articles were identified by reviewing the references cited in the selected articles. 

The gastrointestinal (GI) tract is an uncommon metastatic site for RCC, with only a few reported cases of metastasis from RCC to the rectum and anus. The lack of specific screening guidelines for GI metastasis of RCC highlights the importance of a thorough physical examination and maintaining a strong clinical suspicion for the early detection of metastasis [1-8] (Table 1).

**Table 1:** Summary of the reported cases

Patient	Stage of renal cell carcinoma at the time of diagnosis	Time after initial diagnosis	Chemotherapy/immunotherapy prior to presentation with rectal metastasis	Symptoms at the time of presentation with rectal metastasis	Metastatic status at the time of diagnosis of rectal metastasis
1	Stage 4	9 years	Chemotherapy with interferon, 5-fluorouracil, floxuridine, and thalidomide	Painful bleeding and anal mass	Brain, duodenum, lungs [1]
2	Stage 4	9 months	None	Hematochezia and acute anemia	None [2]
3	Not defined	28 years	Immunotherapy with interleukin-2 and interferon alpha	Hematochezia and acute anemia	Brain, duodenum, lungs [3]
4	Stage 3	7 years	None	Anal mass	Lung, small bowel mesentery [4]
5	Stage 3	9 years	None	Hematochezia, nausea, vomiting, change in stool caliber	Retroperitoneal (left psoas muscle) [5]
6	Not defined	Not described	Chemotherapy with sunitinib	Hematochezia and acute anemia	Lung and lymph nodes [6]
7	Not defined	10 years	Chemotherapy with sunitinib	Incidental rectal mass on colonoscopy	None [7]
8	Stage 3	Same time	None	Painless hematochezia	None [8]

Patient 1

The first patient was a 53-year-old man with a history of stage-4 RCC on the right side for which he had undergone a radical right nephrectomy. He had a history of metastasis to both lungs, metastasis to the brain for which he had undergone resection of the left occipital lobe, as well as chemotherapy and radiation treatment. He had a painful, bleeding anal mass. The mass, which was initially thought to be a thrombosed internal hemorrhoid, was surgically removed. Examination of the resected mass showed nests and sheets of polygonal cells with clear cytoplasm, along with vascularization, all findings consistent with metastasis of clear cell RCC [1].

Patient 2

The second patient was a 55-year-old man who had undergone a right-sided radical nephrectomy for RCC with capsular invasion. Nine months later, he was found to have hematochezia and acute anemia. Colonoscopy and biopsy showed an undifferentiated nodular mass in the rectum, prompting abdominoperineal resection of the rectum. Microscopic analysis of the specimen showed clear cells interspersed with a trabecular growth pattern and vascularization, indicating that RCC was limited to the submucosal layer. Because these microscopic characteristics were highly suggestive of RCC, tumor markers were not determined. The patient subsequently developed metastases to the lungs and bones, ultimately resulting in death [2].

Patient 3

A 70-year-old man with metastatic RCC presented with acute painless hematochezia and anemia. A colonoscopy showed a 2-cm firm rubbery mass in the submucosa adjacent to the dentate line. Biopsy of the mass showed irregular cells with clear cytoplasm, findings consistent with RCC [3].

Patient 4

A 76-year-old man with a history of clear cell RCC and metastasis to the lung, who had undergone right-sided nephroureterectomy seven years earlier, developed an unusual perianal lesion. His small bowel mesentery was evaluated, and the lesion was surgically removed. Pathologic examination showed nests of cells with hyperchromatic nuclei and clear cytoplasm. Immunohistochemistry showed that these cells were positive for vimentin and CD10 [4].

Patient 5

A 67-year-old man with a history of stage-3 left-sided RCC who had undergone left nephrectomy 9 years earlier presented with acute onset of abdominal distention, nausea, vomiting, and hematochezia. A CT and subsequent colonoscopy showed an infiltrative and obstructive mass in the rectosigmoid colon. The patient underwent resection of the sigmoid colon and upper rectum. Analysis of the specimen yielded results consistent with RCC [5].

Patient 6

A 61-year-old man with a history of metastatic RCC to the lung and lymph nodes, who had undergone left nephrectomy followed by treatment with sunitinib, presented with hematochezia and acute anemia. A colonoscopy showed a hemorrhagic pedunculated mass in the upper rectum. Tumor marker analysis showed cells positive for vimentin and negative for CK7 and CK20, both markers for adenocarcinoma, indicating metastasis of RCC to the rectum [6].

Patient 7

A 65-year-old asymptomatic man who had undergone right-sided radical nephrectomy and one round of chemotherapy for clear cell RCC had been screened for colon cancer every one to two years after the completion of the treatment. Ten years after nephrectomy, he was incidentally found to have a 1.2- cm  x  1.5- cm hyperemic protruding mass in the rectum. Microscopic examination of a biopsy sample showed nests of cells with clear cytoplasm and nuclear atypia embedded with vascular tissue cells. Immunohistochemistry showed that these cells were positive for vimentin, pan-cytokeratin pan (AE1/AE3), and paired box (PAX)-8, confirming the diagnosis of metastasis of RCC to the rectum. A PET scan revealed new lesions in bilateral lungs, lymph nodes, and the right ilium along with the upper rectum. The patient was started on treatment with the tyrosine kinase inhibitor sunitinib [7].

Patient 8

A 78-year-old woman with no pertinent prior medical history underwent a colonoscopy for acute onset of painless bleeding from the lower GI tract. Histopathologic evaluation of a rectal polyp revealed a highly vascularized pseudo glandular structure with cells having a clear cytoplasm. Tumor marker examination showed that these cells were positive for pan keratin, CD10, endomysial antibody, and RCC. An abdominal CT revealed a 9-cm left-sided renal mass with a 3-cm left renal vein thrombosis. She later underwent laparoscopic radical resection of the left kidney [8].

Management of RCC primarily focuses on the extent of tissue involvement. Staging of the disease is based on the size of the primary tumor, the involvement of lymph nodes, invasion of surrounding anatomical structures, and metastasis to other organs [9-11]. The primary treatment consists of tumor resection, followed by adjuvant chemotherapy or chemotherapy alone in patients who contradicted for tumor debulking [12,13]. RCC frequently metastasizes to bones, brain, liver, lungs, and lymph nodes [14,15]. The involvement of the GI tract is uncommon [16], whereas RCC seeding of the rectum and anal canal are very rare. This review article describes all patients who have been diagnosed to have RCC metastasizing to the rectum and anal canal to date [1-8].

Rectal bleeding was the most common presentation at the time of diagnosis, with most of these patients found to be at TNM (tumor-node-metastasis) stage three or four. The presentation of RCC metastasis to the rectum and anal canal appears to be similar to that of the primary adenocarcinoma of the rectum. The metastatic disease should, therefore, be considered in the differential diagnosis of elderly patients with a history of RCC who present with fresh bleeding per rectum. The histopathologic analysis is generally sufficient to differentiate primary adenocarcinoma from metastatic RCC. Cells in primary adenocarcinoma usually form a glandular tube-like structure, whereas RCC cells form highly vascularized sinusoidal nest and network, as observed in the patients described in this review.

The optimal time and frequency for primary surveillance of metastasis from RCC have not yet been determined. Generally, patients are at the greatest risk of developing metastasis during the first few years after primary diagnosis. Standardized guidelines have been developed by the American Urological Association and the National Comprehensive Cancer Network to efficiently assess the risk of recurrence of this disease [17-19]. Because these protocols rely more on history taking and physical examination than on imaging methods, this review article emphasizes the need to maintain a high clinical suspicion of metastatic disease in the differential diagnosis of patients with a history of RCC who present with rectal bleeding.

## Conclusions

In this literature review, we summarized several clinical scenarios describing RCC metastasis with the aim of raising awareness among clinicians. RCC is one of the more common cancers of the kidneys and often remains asymptomatic until the disease has progressed significantly. It is standard practice to look for more commonly appearing cancers such as colorectal cancer, adenomas, and polyps in patients with hematochezia or melena. In this literature review, we discussed several clinical cases where gastrointestinal bleeding was deemed secondary to metastatic RCC in the rectum and anal canal. Therefore, RCC metastasis to the rectum and anal canal should be considered in the differential diagnosis of patients with lower GI bleeding, especially patients with the relevant medical history. Appropriate patients should be screened thoroughly by physical examination and imaging modalities.
